# RNSCLC-PRSP software to predict the prognostic risk and survival in patients with resected T_1-3_N_0–2_ M_0_ non-small cell lung cancer

**DOI:** 10.1186/s13040-019-0205-0

**Published:** 2019-08-23

**Authors:** Yunkui Zhang, YaoChen Li, Rongsheng Zhang, Yujie Zhang, Haitao Ma

**Affiliations:** 1 Department of Thoracic Surgery, The First Affiliated Hospital of Soochow University, No. 899 Pinghai Road, Suzhou, 215006 China; 2 Department of Thoracic Surgery, Shanxi Tumor Hospital, No. 3 Zhigongxin Street, Taiyuan, 030013 China; 3 The Central Laboratory of Cancer Hospital of Shantou University Medical College, Guangdong Provincial Key Laboratory on Breast Cancer Diagnosis and Treatment Research, No. 7 Raoping Road, Shantou, 515031 China

**Keywords:** Resected non-small cell lung cancer, Prognostic risk prediction, Survival prediction, Prognostic index, Software

## Abstract

**Background:**

The clinical outcomes of patients with resected T_1-3_N_0–2_M_0_ non-small cell lung cancer (NSCLC) with the same tumor-node-metastasis (TNM) stage are diverse. Although other prognostic factors and prognostic prediction tools have been reported in many published studies, a convenient, accurate and specific prognostic prediction software for clinicians has not been developed. The purpose of our research was to develop this type of software that can analyze subdivided T and N staging and additional factors to predict prognostic risk and the corresponding mean and median survival time and 1–5-year survival rates of patients with resected T_1-3_N_0–2_M_0_ NSCLC.

**Results:**

Using a Cox proportional hazard regression model, we determined the independent prognostic factors and obtained a prognostic index (PI) eq. PI = ∑_βixi_.

=0.379X_1_–0.403X_2_–0.267X_51_–0.167X_61_–0.298X_62_ + 0.460X_71_ + 0.617X_72_–0.344X_81_–0.105X_91_–0.243X_92_ + 0.305X_101_ + 0.508X_102_ + 0.754X_103_ + 0.143X_111_ + 0.170X_112_ + 0.434X_113_–0.327X_122_–0.247X_123_ + 0.517X_133_ + 0.340X_134_ + 0.457X_143_ + 0.419X_144_ + 0.407X_145_. Using the PI equation, we determined the PI value of every patient. According to the quantile of the PI value, patients were divided into three risk groups: low-, intermediate-, and high-risk groups with significantly different survival rates. Meanwhile, we obtained the mean and median survival times and 1–5-year survival rates of the three groups. We developed the RNSCLC-PRSP software which is freely available on the web at http://www.rnsclcpps.com with all major browsers supported to determine the prognostic risk and associated survival of patients with resected T_1-3_N_0–2_ M_0_ non-small cell lung cancer.

**Conclusions:**

After prognostic factor analysis, prognostic risk grouping and corresponding survival assessment, we developed a novel software program. It is practical and convenient for clinicians to evaluate the prognostic risk and corresponding survival of patients with resected T_1-3_N_0–2_M_0_ NSCLC. Additionally, it has guiding significance for clinicians to make decisions about complementary treatment for patients.

**Electronic supplementary material:**

The online version of this article (10.1186/s13040-019-0205-0) contains supplementary material, which is available to authorized users.

## Background

Lung cancer is the first leading cause of cancer death among men and the second leading cause of cancer death for women worldwide [1]. At present, the eighth edition of non-small cell lung cancer (NSCLC) tumor-node-metastasis (TNM) staging system developed and validated by the International Association for the Staging of Lung Cancer (IASLC) project is considered to be the most significant prognostic predictor and the main guider of postoperative supplementary treatment [2]. The following factors were incorporated into the IASLC system: histological grade, gender, age, and performance status. No molecular prognostic factors are used in the clinic because of the lack of cross-validation, Even the new biomarker programmed cell death protein 1 ligand (PD-L1) is a predictive marker of good response to immunotherapy drugs but poor prognostic indicator of survival [3]. However, clinicians know that the outcomes are diverse among resected NSCLC patients with the same TNM stage and other similar clinical features. Some die early after surgical treatment, while some remain alive, even living longer than expected. Therefore, for clinicians, subgroups of T and N staging and other more clinicopathological features should be considered in prognostic risk and survival prediction.

Recently, there have been many studies on the prognostic factors for patients with resected NSCLC [4–7]. Prognostic factors can be divided into clinical factors, tumor-related factors and treatment-related factors. TNM stage, gender, age, number of examined regional lymph nodes (NELNs), number of positive regional lymph nodes (NPLNs), surgery type, histological grade, histology, and marital status have been reported to be prognostic factors for patients with resected NSCLC [8–22]. There have been few studies on T and N staging subgroups as prognostic factors. Meanwhile, some prognostic prediction tools, such as prognostic nomograms, scores, and survival models for patients with resected NSCLC, have been reported in many published studies [23–27]. Unfortunately, for clinicians who are busy in clinical work, it is inconvenient to use the TNM stage system and tools for which the results were inaccurate and vague. Therefore, we aimed to develop software that can conveniently, specifically, accurately predict the prognostic risk and survival of patients with T_1-3_N_0–2_M_0_ NSCLC. In the process of building the model, T and N staging subgroups and other more clinical features were analyzed as prognostic factors.

### Implementation

**Fig. 1:**
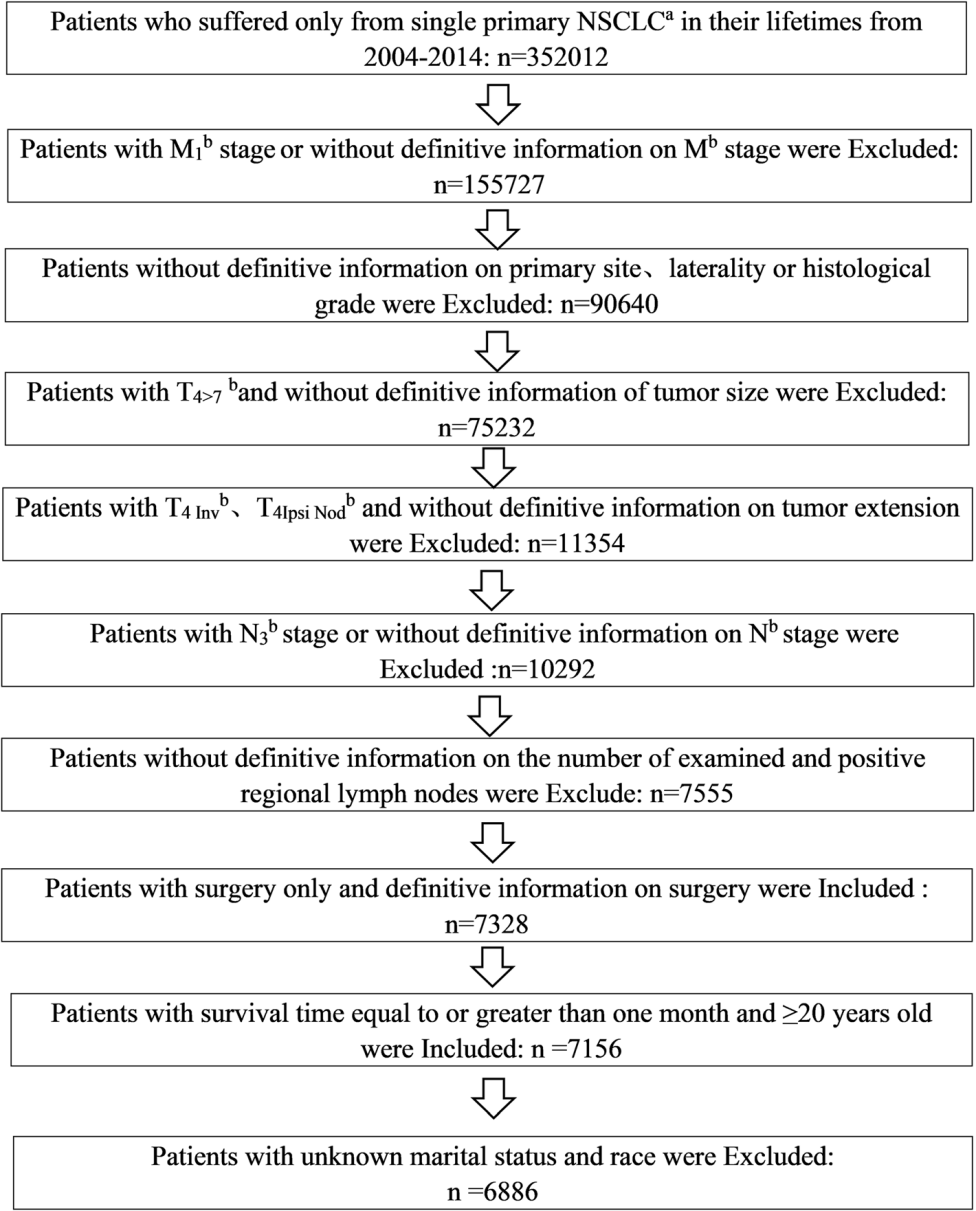
According to the inclusion and exclusion criteria, the flow chart of screening patients. **a** NSCLC: non-small cell lung cancer. **b** According to the eighth edition of American Joint Committee on Cancer (AJCC)/ Union for International Cancer Control (UICC) stage classification for NSCLC

We collected information on patients from the Surveillance, Epidemiology, and End Results (SEER) database, which provides cancer statistics for U.S. patients. In this study, 6886 patients were obtained. Eligibility criteria included the following: [1] histological diagnosis of NSCLC; [2] suffering from only single primary NSCLC in their lifetime and had NSCLC between 2004 and 2014; [3] received resection only; [4] had definitive surgical information; [5] survival time equal to or greater than one month; and [6] ≥20 years old. Moreover, the following criteria were used to exclude patients from the study: [1] M_1_ stage or without definitive information on M stage; [2] without definitive information on primary site, laterality or histological grade; [3] with T_4>7_ and without definitive information on tumor size; [4] with T_4 Inv_, T_4 Ipsi Nod_ and without definitive information on tumor extension; [5] with N_3_ stage or without definitive information on N stage; [6] without definitive information on the number of examined and positive regional lymph nodes; [7] unknown marital status and race. Figure 1 shows the flow chart of the process used to screen patients according to the inclusion and exclusion criteria. Clinicopathological characteristics and follow-up information were collected, as shown in Table 1, including gender, age, laterality, race, N stage, NELNs, NPLNs, surgery type, primary site, histological grade, histology, marital status, tumor extension, tumor size, survival months and status.

**Table 1:** The clinicopathological characteristics of patients with resected T_1-3_N_0 − 2_ M_0_ NSCLC

Characteristics	Number of patients
All patients	6886 (100%)
Gender	
Male	3363 (48.8%)
Female	3523 (51.2%)
Age	
≤65	2964 (43.0%)
> 65	3922 (57.0%)
Laterality	
Right	3958 (57.5%)
Left	2928 (42.5%)
Race	
White	5770 (83.8%)
Black	589 (8.6%)
Others	527 (7.6%)
N stage ^a^	
N_0_	4578 (66.5%)
N_1_	1228 (17.8%)
N_2_	1080 (15.7%)
NELNs	
N ≤ 6	2495 (36.2%)
6<N ≤ 12	2272 (33.0%)
N>12	2119 (30.8%)
NPLNs	
N = 0	4658 (67.6%)
1 ≤ N ≤ 3	1623 (23.6%)
N ≥ 4	605 (8.8%)
Surgery type	
SLET	599 (8.7%)
LET	5748 (83.5%)
PET	539 (7.8%)
Primary site	
UL	4125 (60.0%)
ML	414 (6.0%)
LL	2213 (32.1%)
Others	134 (1.9%)
Histological grade	
I	816 (11.9%)
II	3158 (45.9%)
III	2766 (40.2%)
IV	146 (2.0%)
Histology	
AC	3013 (43.8%)
S	1629 (23.7%)
ASC	206 (3.0%)
BAA	220 (3.2%)
Others	1818 (26.3%)
Marital status	
Single (never married)	801 (11.6%)
Married	4115 (59.8%)
Divorced	854 (12.4%)
Widowed	1017 (14.8%)
Others	99 (1.4%)
Tumor extension ^a^	
T_1a ss_	729 (10.6%)
T_2 Visc PI_	4078 (59.2%)
T_2 Centr_	1366 (19.8%)
T_3 Inv_	68 (1.0%)
T_3 Satell_	645 (9.4%)
Tumor size ^a^	
T_1a ≤ 1(T ≤ 1)_	193 (2.8%)
T_1b>1–2(1<T ≤ 2)_	1573 (22.8%)
T_1c>2–3(2<T ≤ 3)_	1932 (28.1%)
T_2a>3–4(3<T ≤ 4)_	1449 (21.0%)
T_2b>4–5(4<T ≤ 5)_	865 (12.6%)
T_3>5–7(5<T ≤ 7)_	874 (12.7%)
Survival status	
Dead	2443 (35.5%)
Alive	4443 (64.5%)

Abbreviations: *NELNs* Number of examined regional lymph nodes, *NPLNs* Number of positive regional lymph nodes, *SLET* sublobectomy, *LET* Lobectomy, *PET* Pneumonectomy, *UL* Upper lobe, *ML* Middle lobe, *LL* Lower lobe, *I* Well differentiated, *II* Moderately differentiated, *III* Poorly differentiated, *IV* Undifferentiated, *AC* Adenocarcinoma, *S* Squamous carcinoma, *ASC* Adenosquamous carcinoma, *BAA* Bronchioalveolar adenocarcinoma

^a^ According to the eighth edition of the AJCC/UICC stage classification for NSCLC

First, In this data set, approximately 70% of patients were randomly assigned to the training set (resulting in 4821 patients), while the remaining patients comprised the test set (resulting in 2065 patients). The training set was used to build the model, and the test set was used to verify the model. Second, based on the training set, the Cox proportional hazard regression model was used to identify independent prognostic factors and their model coefficients. Third, we obtained a prognostic index (PI) equation, which is the value of each independent prognostic factor and the sum of the corresponding regression coefficient product. Fourth, according to the quantile of the PI value, patients were divided into three risk groups: the low-, intermediary-, and high-risk groups with significantly different survival rates according to Kaplan-Meier analysis and log-rank test. Meanwhile, we obtained the mean and median survival times and 1–5-year survival rates of the three risk groups. We used a test set to verify the model. Finally, we developed a software program named RNSCLC-PRSP to predict the prognostic risk and survival of patients with resected T_1-3_N_0–2_M_0_ non-small cell lung cancer by selecting their clinicopathological features. The software is freely available on the web at http://www.rnsclcpps.com with all major browsers supported. Clinicians register and log in and then they select the clinicopathological characteristics of patients, and the prognostic risk and survival outcome are predicted.

We used SPSS (version 16.0) software (Inc, Chicago, IL, USA) for all statistical calculations, and P<0.05 was considered to be significant. Meanwhile, the tree model analysis method was also used to rank the importance of each variable for prediction,

## Results

### Univariate analysis of prognostic factors

Variables codes and assignment methods of clinicopathological characteristics are provided in the Additional file 1: Table S1. After the univariate analysis, the result of which are presented in Table 2, gender, age, N stage, NELNs, NPLNs, surgery type, primary site, histological grade, histology, marital status, tumor extension, and tumor size were significant prognostic factors (P<0.05).

**Table 2:** Univariate analysis of the Cox proportional hazard regression model of resected T_1-3_N_0 − 2_ M_0_ NSCLC

Factors	Variates	b	SE	RR	95%CI	*P*
Gender	X_1_	0.363	0.049	1.438	1.307~1.582	< 0.001
Age	X_2_	−0.354	0.050	0.702	0.637~0.774	< 0.001
Laterality	X_3_	− 0.009	0.049	0.991	0.900~1.091	0.858
Race (as dummy variables)	X_4_					
Others				1.0		
White	X_41_	0.159	0.102	1.172	0.961~1.430	0.118
Black	X_42_	0.172	0.130	1.188	0.920~1.532	0.186
N stage ^a^ (as dummy variables)	X_5_					
N_0_	X_50_	−0.858	0.060	0.424	0.377~0.477	< 0.001
N_1_	X_51_	−0.220	0.071	0.803	0.699~0.922	0.002
NELNs (as dummy variables)	X_6_					
N ≤ 6				1.0		
6<N ≤ 12	X_61_	−0.121	0.058	0.886	0.790~0.993	0.038
N>12	X_62_	−0.046	0.059	0.956	0.852~1.072	0.438
NPLNs (as dummy variables)	X_7_					
N = 0				1.0		
1 ≤ N ≤ 3	X_71_	0.698	0.054	2.009	1.808~2.234	< 0.001
N ≥ 4	X_72_	0.862	0.074	2.367	2.046~2.739	< 0.001
Surgery type (as dummy variables)	X_8_					
SLET				1.0		
LET	X_81_	−0.242	0.086	0.785	0.664~0.929	0.005
PET	X_82_	0.088	0.109	1.092	0.882~1.353	0.420
Primary site (as dummy variables)	X_9_					
Others	X_90_	−0.239	0.160	0.788	0.576~1.078	0.136
UL	X_91_	−0.114	0.052	0.892	0.806~0.987	0.028
ML	X_92_	−0.273	0.114	0.761	0.609~0.952	0.017
Histological grade (as dummy variables)	X_10_					
I				1.0		
II	X_101_	0.551	0.096	1.736	1.437~2.097	< 0.001
III	X_102_	0.834	0.095	2.301	1.909~2.775	< 0.001
IV	X_103_	0.998	0.161	2.712	1.977~3.722	< 0.001
Histology (as dummy variables)	X_11_					
Others				1.0		
AC	X_111_	0.197	0.063	1.217	1.076~1.377	0.002
S	X_112_	0.379	0.068	1.462	1.280~1.669	< 0.001
ASC	X_113_	0.588	0.134	1.801	1.385~2.342	< 0.001
BAA	X_114_	−0.304	0.156	0.738	0.543~1.002	0.051
Marital status (as dummy variables)	X_12_					
Others	X_120_	−0.057	0.216	0.945	0.618~1.444	0.793
Single (never married)	X_121_	−0.258	0.092	0.773	0.645~0.926	0.005
Married	X_122_	−0.286	0.066	0.751	0.660~0.855	< 0.001
Divorced	X_123_	−0.290	0.090	0.749	0.627~0.894	0.001
Tumor extension^a^ (as dummy variables)	X_13_					
T_1a ss_				1.0		
T_2 Visc PI_	X_131_	0.114	0.082	1.121	0.954~1.317	0.166
T_2 Centr_	X_132_	0.353	0.085	1.424	1.206~1.680	< 0.001
T_3 Inv_	X_133_	0.809	0.196	2.247	1.529~3.300	< 0.001
T_3 Satell_	X_134_	0.514	0.092	1.672	1.395~2.002	< 0.001
Tumor size^a^ (as dummy variables)	X_14_					
T_1a ≤ 1(T ≤ 1)_				1.0		
T_1b>1–2(1<T ≤ 2)_	X_141_	0.016	0.172	1.016	0.725~1.425	0.927
T_1c>2–3(2<T ≤ 3)_	X_142_	0.361	0.169	1.434	1.030~1.997	0.033
T_2a>3–4(3<T ≤ 4)_	X_143_	0.584	0.170	1.793	1.286~2.501	0.001
T_2b>4–5(4<T ≤ 5)_	X_144_	0.585	0.174	1.794	1.276~2.523	0.001
T_3>5–7(5<T ≤ 7)_	X_145_	0.664	0.174	1.943	1.382~2.732	< 0.001

Abbreviations: *B* Regression coefficient, *SE* Standard error, *RR* Relative risk, *CI* Confidence interval, *NELNs* Number of examined regional lymph nodes, *NPLNs* Number of positive regional lymph nodes, *SLET* Sublobectomy, *LET* Lobectomy, *PET* Pneumonectomy, *UL* Upper lobe, *ML* Middle lobe, *LL* Lower lobe, *I* Well differentiated, *II* Moderately differentiated, *III* Poorly differentiated, *IV* Undifferentiated, *AC* Adenocarcinoma, *S* Squamous carcinoma, *ASC* Adenosquamous carcinoma, *BAA* Bronchioalveolar adenocarcinoma

^a^ According to the eighth edition AJCC/UICC stage classification for NSCLC.

### Multivariate analysis of prognostic factors

By multivariate analysis of prognostic factors, the results of which are shown in Table 3, gender, age, N_1_ stage, NELNs (6<N ≤ 12, N>12), NPLN (1 ≤ N ≤ 3, N ≥ 4), lobectomy (LET), primary site (UL, ML), histological grade (II, III, IV), histology (AC, S, ASC), marital status (married, divorced), tumor extension (T_3 Inv_, T_3 Satell_), and tumor size (T_2a>3–4(3<T ≤ 4)_, T_2b>4–5(4<T ≤ 5)_, T_3>5–7(5<T ≤ 7)_) were identified as independent prognostic factors.

**Table 3:** Multivariate analysis of the Cox proportional hazard regression model of resected T_1-3_N_0 − 2_ M_0_ NSCLC

Factors	Variates	b	SE	RR	95%CI	*P*
Gender	X_1_	0.379	0.053	1.460	1.317~1.620	< 0.001
Age	X_2_	−0.403	0.054	0.668	0.601~0.743	< 0.001
N stage						0.001
N_0_	X_50_	−0.372	0.195	0.689	0.470~1.010	0.056
N_1_	X_51_	−0.267	0.075	0.766	0.661~0.886	< 0.001
NELNs						< 0.001
6<N ≤ 12	X_61_	−0.167	0.060	0.846	0.751~0.952	0.006
N>12	X_62_	−0.298	0.064	0.742	0.655~0.841	< 0.001
NPLNs						0.003
1 ≤ N ≤ 3	X_71_	0.460	0.197	1.583	1.077~2.328	0.019
N ≥ 4	X_72_	0.617	0.203	1.854	1.245~2.762	0.002
Surgery type						0.001
LET	X_81_	−0.344	0.090	0.709	0.595~0.845	< 0.001
PET	X_82_	−0.245	0.127	0.783	0.611~1.003	0.053
Primary site						0.035
Others	X_90_	−0.308	0.172	0.735	0.525~1.029	0.073
UL	X_91_	−0.105	0.053	0.900	0.811~0.999	0.047
ML	X_92_	−0.243	0.116	0.784	0.625~0.983	0.035
Histological grade						< 0.001
II	X_101_	0.305	0.100	1.356	1.114~1.651	0.002
III	X_102_	0.508	0.101	1.663	1.364~2.027	< 0.001
IV	X_103_	0.754	0.167	2.126	1.532~2.950	< 0.001
Histology						0.011
AC	X_111_	0.143	0.066	1.153	1.013~1.313	0.031
S	X_112_	0.170	0.073	1.186	1.028~1.368	0.019
ASC	X_113_	0.434	0.137	1.544	1.181~2.019	0.001
BAA	X_114_	−0.030	0.160	0.970	0.709~1.328	0.851
Marital status						< 0.001
Others	X_120_	0.016	0.221	1.016	0.659~1.566	0.944
Single (never married)	X_121_	−0.133	0.098	0.875	0.723~1.060	0.172
Married	X_122_	−0.327	0.071	0.721	0.628~0.829	< 0.001
Divorced	X_123_	−0.247	0.094	0.781	0.650~0.938	0.008
Tumor extension^a^						< 0.001
T_2 Visc PI_	X_131_	−0.115	0.087	0.892	0.752~1.056	0.185
T_2 Centr_	X_132_	0.025	0.092	1.025	0.856~1.229	0.786
T_3 Inv_	X_133_	0.517	0.204	1.678	1.125~2.500	0.011
T_3 Satell_	X_134_	0.340	0.095	1.405	1.167~1.692	< 0.001
Tumor size^a^						< 0.001
T_1b>1–2(1<T ≤ 2)_	X_141_	0.025	0.174	1.025	0.729~1.442	0.886
T_1c>2–3(2<T ≤ 3)_	X_142_	0.260	0.171	1.297	1.927~1.815	0.129
T_2a>3–4(3<T ≤ 4)_	X_143_	0.457	0.174	1.580	1.122~2.223	0.009
T_2b>4–5(4<T ≤ 5)_	X_144_	0.419	0.179	1.520	1.069~2.161	0.020
T_3>5–7(5<T ≤ 7)_	X_145_	0.407	0.180	1.502	1.055~2.140	0.024

Abbreviations: *b* Regression coefficient, *SE* Standard error, *RR* Relative risk, *CI* Confidence interval, *NELNs* Number of examined regional lymph nodes, *NPLNs* Number of positive regional lymph nodes, *LET* Lobectomy, *PET* Pneumonectomy, *UL* Upper lobe, *ML* Middle lobe, *II* Moderately differentiated, *III* Poorly differentiated, *IV* Undifferentiated, *AC* Adenocarcinoma, *S* Squamous carcinoma, *ASC* Adenosquamous carcinoma, *BAA* Bronchioalveolar adenocarcinoma

^a^ According to the eighth edition AJCC/UICC stage classification for NSCLC

### The tree model analysis

The tree model analysis method was used to rank the importance of each variable for prediction. The results are shown in Table 4. The third column is standardized importance. The first 12 variables were selected into the model, which was consistent with the Cox regression results.

**Table 4:** The importance of each variable for prediction

Variable	Importance	Standard importance
Tumor extension	0.045	100.0%
N stage	0.016	35.5%
NPLNs	0.015	33.1%
Histology	0.007	15.4%
Surgery type	0.007	14.9%
Age	0.005	11.5%
Gender	0.005	10.6%
Histological grade	0.004	9.8%
Primary site	0.002	4.9%
Marital status	0.002	3.6%
NELNs	0.001	2.8%
Tumor size	0.001	2.2%
Race	0.001	1.4%
Laterality	0.000	1.0%

Abbreviations: *NPLNs* Number of positive regional lymph nodes, *NELNs* Number of examined regional lymph nodes, *CRT* Classification regression tree

Method: CRT

Y: survival status

^a^ According to the eighth edition AJCC/UICC stage classification for NSCLC

### Prognostic risk model construction and software development

Using the Cox proportional hazard regression model, we obtained the PI equation, PI = ∑_βixi_.

**Fig. 2:**
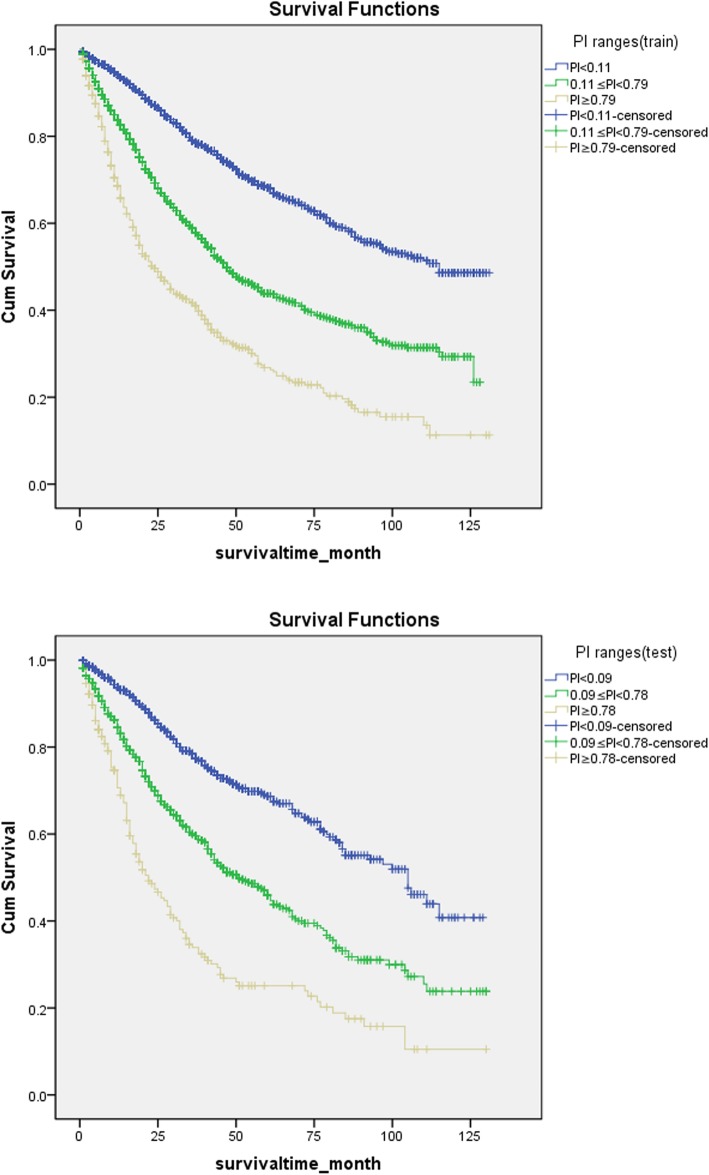
Kaplan-Meier survival curve of PI ranges

=0.379X_1_–0.403X_2_–0.267X_51_–0.167X_61_–0.298X_62_ + 0.460X_71_ + 0.617X_72_–0.344X_81_–0.105X_91_–0.243X_92_ + 0.305X_101_ + 0.508X_102_ + 0.754X_103_ + 0.143X_111_ + 0.170X_112_ + 0.434X_113_–0.327X_122_–0.247X_123_ + 0.517X_133_ + 0.340X_134_ + 0.457X_143_ + 0.419X_144_ + 0.407X_145_. Using the PI equation, we obtained the PI value of every patient. As shown in Table 5, we obtained PI ranges for the training and test sets. According to the quantile of the PI value, we divided patients in the training and test sets into three risk groups. The three risk groups were divided based on the PI values as follow: 0~50%, 50~90%, and 90 + %. The quantiles are divided into low-, intermediary-, and high-risk groups. We obtained three risk groups and their corresponding mean and median survival times and 1–5-year survival rates of the training and test sets (Tables 6 and 7, respectively). Using K-M curves and log-rank tests, we found that, from the low-, intermediate- and high-risk groups, the survival rates of the training and test sets were worse stepwise (*P*<0.001) (Fig. 2). Through the test set verification, the model effect is good.

**Table 5:** PI ranges of the training and test sets

	PI-train	PI-test
20%	−0.37	−0.35
40%	−0.03	− 0.05
50%	0.11	0.09
60%	0.26	0.23
80%	0.58	0.55
90%	0.79	0.78

**Table 6:** (training-set) Three risk groups and their corresponding mean and median survival times and 1–5-year survival rates

Groups	PI ranges	Survival time (months)		Survival rates (%)				
		Mean	Median	1 year	2 year	3 year	4 year	5 year
Low risk	PI≤0.11	90.16	115.0	94.1	87.0	79.0	73.5	68.2
Intermediate risk	0.11<PI<0.79	63.86	47.0	83.9	69.3	58.9	49.1	43.8
High risk	PI≥0.79	42.93	24.0	68.6	49.7	41.6	32.6	26.8

**Table 7:** (test-set) Three risk groups and their corresponding mean and median survival times and 1–5-years survival rates

Groups	PI ranges	Survival time (months)		Survival rates (%)				
		Mean	Median	1 year	2 year	3 year	4 year	5 year
Low risk	PI≤0.09	86.80	105.00	93.8	86.2	78.4	72.1	68.7
Intermediate risk	0.09<PI<0.78	63.09	51.00	84.5	69.9	60.0	51.2	45.9
High risk	PI≥0.78	40.55	22.00	70.6	47.3	33.9	26.8	25.1

We developed a software named RNSCLC-PRSP to predict the prognostic risk and survival of patients with resected T_1-3_N_0–2_M_0_ NSCLC.

## Discussion

We have invented a novel tool to predict the prognosis of patients with resected T_1-3_N_0–2_M_0_ NSCLC. We determined the independent risk factors and obtained prognostic risk models and risk groups and their corresponding survival times. This paper highlights that comprehensive and further refined analysis that is capable with the incorporation of clinical pathological factors to predict prognosis of resected T_1-3_N_0–2_M_0_ NSCLC.

To access the program, clinicians can enter the url http://www.rnsclcpps.com in a browse to reach the login screen of the software. At the bottom of interface is a brief introduction of the software and an explanation of the relevant abbreviations. Above the interface is the login box. New users can click the button of register on the login box to register. After successful registration, users can click the button to return to the login, enter the username and password, click the button to login and enter the software interface. The first line of interface is titled Prognostic risk and survival prediction software RNSCLC-PRSP for resected T_1-3_N_0–2_M_0_ NSCLC (according to the eighth edition AJCC/UICC stage classification). Operational tips (notes) are located under the title, under the note is an explanation of the relevant abbreviations, and there are alternative options located under the abbreviations. According to the note and explanation of abbreviations, clinicians first need to determine the clinicopathological characteristics of patients. Taking a resected T_1-3_N_0–2_M_0_ (according to the eighth edition of AJCC/UICC stage classification) non-small cell lung cancer patient as an example, the clinicopathological characteristics of a representative patient were gender (man), age (≤65), N stage (N_0_), NELNs (N>12) ,NPLNs (N ≥ 4) ,surgery type (LET) ,primary site (UL) ,histological grade (III) ,histology (S) ,marital status (married) ,tumor extension (T_3 Inv_) ,tumor size (T_2b>4–5(4<T ≤ 5)_). For these clinicopathological characteristics, clinicians can choose the appropriate response for each factor. If there are no corresponding options, clinicians should choose none and then click the button to submit their entry, and the prognostic risk and survival prediction results will be shown on the next page. Here are the prognostic and prediction results for the representative patients: high-risk group, PI value is PI≥0.79, mean and median survival time are 42.93 and 24.0 months respectively, and the 1–5 year survival rates are 68.6, 49.7, 41.6, 32.6, 26.8% respectively.

The RNSCLC-PRSP software we have developed is based on the actual needs of clinicians predicting the prognosis of patients with resected NSCLC. Clinicians are very busy in clinical work; meanwhile, the prognosis of resected NSCLC patients is affected by many factors. There is no more time for clinicians to evaluate every factor to obtain a more accurate prognosis. We provide quantitative and relative analysis software, and clinicians can conveniently and swiftly get every patient’s prognostic risk and survival calculated accurately just by choosing some of the clinicopathological features. The RNSCLC-PRSP software would be gladly accepted by clinicians. At present, there have been no relative prognostic predictive software programs for resected T_1-3_N_0–2_M_0_ NSCLC. Pilotto S et al. developed clinicopathological prognostic nomograms for resected squamous cell lung cancer, Based on clinicopathological factors including age, T descriptor (according to the seventh edition of the TNM classification), lymph node status, and grading in the model. Every patient was assigned a prognostic score [28]. Francesco Guerrera et al. designed a prognostic model predicting 5-year survival after surgical resection for stage I non-small cell lung cancer based on clinical, pathological and surgical covariates [25]. Compared to the above two tools, our software analysis includes more clinicopathological features and more detail for more patients with resected non-small cell lung cancer and our novel software is more convenient and practical for clinicians.

Although we have established predictive software using relative prognostic factors, we may need to analyze more clinicopathological factors to improve the software. Thus, further research will be conducted. The potential valuable prognostic prediction factors such as smoking status, performance status, comorbidity, molecular biological factors, biochemical and biomarker test results, lung function, tumor vascular or lymphatic invasion, surgical method (minimally invasive or open), and surgery margins, were not able to be determined or researched in more recent database. However, with the expansion of databases, further research will be carried out, and our software can be updated and improved to provide better service.

## Conclusions

Using the SEER database and the Cox proportional hazard model, we identified the independent prognostic factors and corresponding PI value of patients with resected T_1-3_N_0–2_M_0_ NSCLC. According to different PI ranges, three prognostic risk groups (the low-, intermediate-, high-risk groups) were determined, and their corresponding survival times were obtained. We developed the RNSCLC-PRSP software for clinicians to conveniently and practically predict the prognosis of patients with resected T_1-3_N_0–2_M_0_ NSCLC to guide further treatment. We have shown that the software we have developed opens a new predictive method in this field.

## Availability and requirements

Project name: My bioinformatics project.

Project home page: http://www.rnsclcpps.com

Operating system(s): Platform independent.

Programming language: Java.

Other requirements: no.

License: no.

Any restrictions to use by non-academics: no.

## Additional file

Additional file 1: **Table S1.** Variable codes and assignment methods of Cox proportional hazard regression model analysis of resected T_1-3_N_0-2_ M_0_ NSCLC. (DOCX 22 kb)

## Data Availability

The datasets used and/or analyzed during the current study are available from the corresponding author on reasonable request.

## Abbreviations

AC: Adenocarcinoma
AJCC: American Joint Committee on Cancer
ASC: Adenosquamous carcinoma
ATS: American Thoracic Society
B: Regression coefficient
BAA: Bronchioalveolar adenocarcinoma
CI: Confidence interval
DFS: Disease free survive
ERS: European Respiratory Society
I: Well differentiated
IASLC: International Association for the Study of Lung Cancer
II: Moderately differentiated
III: Poorly differentiated
IV: Undifferentiated
LET: Lobectomy
LL: Lower lobe
ML: Middle lobe
NELNs: Number of examined regional lymph nodes
NPLNs: Number of positive regional lymph nodes
NSCLC: Non-small cell lung cancer
OS: Overall survival
PD-L1: Programmed cell death protein 1 ligand
PET: Pneumonectomy
PI: Prognosis Index
RR: Relative risk
S: Squamous carcinoma
SE: Standard error
SEER: The Surveillance Epidemiology and End Results
SLET: Sublobectomy
TNM: Tumor Node Metastasis
UICC: Union for International Cancer Control
UL: Upper lobe
