# Supplementary material for: RNSCLC-PRSP software to predict the prognostic risk and survival in patients with resected T1-3N0–2 M0 non-small cell lung cancer
Source: BioData Min. 2019 Aug 23;12:17. doi: 10.1186/s13040-019-0205-0 (PMC6708148; doi:10.1186/s13040-019-0205-0)
Supplement: Supplementary file 1 — Table S1. Variable codes and assignment methods of Cox proportional hazard regression model analysis of resected T1-3N0-2 M0 NSCLC. (DOCX 22 kb) [file 13040_2019_205_MOESM1_ESM.docx]

Additional file 1

**Variable codes and assignment methods of clinicopathological characteristics (Table 7).**

**Table S1**.Variable codes and assignment methods of Cox proportional hazard regression model analysis of resected T_1-3_N_0-2_ M_0_ NSCLC.

| Codes | Variables | Assignment methods | |
| --- | --- | --- | --- |
| X_1_ | Gender | Male: 1 | Female: 0 |
| X_2_ | Age | ≤65: 1 | >65: 0 |
| X_3_ | Laterality | Right: 1 | Left: 0 |
| X_4_ | Race |  |  |
| X_41_ | White | Yes: 1 | No: 0 |
| X_42_ | Black | Yes: 1 | No: 0 |
| X_5_ | N stage ^a^ |  |  |
| X_50_ | N_0_ | Yes: 1 | No: 0 |
| X_51_ | N_1_ | Yes: 1 | No: 0 |
| X_6_ | NELN |  |  |
| X_61_ | 6˂N≤12 | Yes: 1 | No: 0 |
| X_62_ | N˃12 | Yes: 1 | No: 0 |
| X_7_ | NPLN |  |  |
| X_71_ | 1≤N≤3 | Yes: 1 | No: 0 |
| X_72_ | N≥4 | Yes: 1 | No: 0 |
| X_8_ | Surgerytype |  |  |
| X_81_ | LET | Yes: 1 | No: 0 |
| X_82_ | PET | Yes: 1 | No: 0 |
| X_9_ | Primary Site |  |  |
| X_90_ | others | Yes: 1 | No: 0 |
| X_91_ | UL | Yes: 1 | No: 0 |
| X_92_ | ML | Yes: 1 | No: 0 |
| X_10_ | Histological Grade |  |  |
| X_101_ | Ⅱ | Yes: 1 | No: 0 |
| X_102_ | Ⅲ | Yes: 1 | No: 0 |
| X_103_ | Ⅳ | Yes: 1 | No: 0 |
| X_11_ | Histology |  |  |
| X_111_ | AC | Yes: 1 | No: 0 |
| X_112_ | S | Yes: 1 | No: 0 |
| X_113_ | ASC | Yes: 1 | No: 0 |
| X_114_ | BAA | Yes: 1 | No: 0 |
| X_12_ | Marital Status |  |  |
| X_120_ | others | Yes: 1 | No: 0 |
| X_121_ | single (never married) | Yes: 1 | No: 0 |
| X_122_ | married | Yes: 1 | No: 0 |
| X_123_ | divorced | Yes: 1 | No: 0 |
| X_13_ | Tumor Extension ^a^ |  |  |
| X_131_ | T_2 Visc PI_ | Yes: 1 | No: 0 |
| X_132_ | T_2 Centr_ | Yes: 1 | No: 0 |
| X_133_ | T_3 Inv_ | Yes: 1 | No: 0 |
| X_134_ | T_3 Satell_ | Yes: 1 | No: 0 |
| X_14_ | Tumor Size ^a^ |  |  |
| X_141_ | T_1b˃1-2_(1˂T≤2) | Yes: 1 | No: 0 |
| X_142_ | T_1c˃2-3_(2˂T≤3) | Yes: 1 | No: 0 |
| X_143_ | T_2a˃3-4_(3˂T≤4) | Yes: 1 | No: 0 |
| X_144_ | T_2b˃4-5_(4˂T≤5) | Yes: 1 | No: 0 |
| X_145_ | T_3˃5-7_(5˂T≤7) | Yes: 1 | No: 0 |

1. According to the eighth edition of the AJCC/UICC stage classification for NSCLC.

Abbreviations: NELNs=number of examined regional lymph nodes; NPLNs=number of positive regional lymph nodes; SLET=sublobectomy; LET=lobectomy; PET=pneumonectomy; UL=upper lobe; ML=middle lobe; LL=lower lobe; Ⅰ=well differentiated; Ⅱ=moderately differentiated; Ⅲ=poorly differentiated; Ⅳ=undifferentiated; AC=adenocarcinoma; S=squamous carcinoma; ASC=adenosquamous carcinoma; BAA=bronchioalveolar adenocarcinoma

**About the PI Value**

PI is the sum of arithmetic product of variable assignment and its corresponding regression coefficients. For positive regression coefficients, if the numerical value is large, the PI presents added value, indicate an adverse prognosis. If the numerical value is small, the PI presents a favorable prognosis. For the negative regression coefficients, there are adverse results. With increasing PI, the survival rates decease gradually.
